# Inhibition of Histone Deacetylase (HDAC) Enhances Checkpoint Blockade Efficacy by Rendering Bladder Cancer Cells Visible for T Cell-Mediated Destruction

**DOI:** 10.3389/fonc.2020.00699

**Published:** 2020-05-15

**Authors:** Brianna Burke, Catherine Eden, Cynthia Perez, Alex Belshoff, Spencer Hart, Lourdes Plaza-Rojas, Michael Delos Reyes, Kushal Prajapati, Christina Voelkel-Johnson, Elizabeth Henry, Gopal Gupta, José Guevara-Patiño

**Affiliations:** ^1^Department of Surgery and Cancer Biology, Loyola University Chicago, Chicago, IL, United States; ^2^Department of Urology, Loyola University Medical Center, Maywood, IL, United States; ^3^Department of Microbiology and Immunology, Medical University of South Carolina, Charleston, SC, United States; ^4^Department of Oncology, Loyola University Medical Center, Maywood, IL, United States

**Keywords:** HDAC, bladder cancer, T cells, NKG2D, anti-PD1, immune evasion

## Abstract

Inhibitory checkpoint blockade therapy is an immunomodulatory strategy that results in the restoration of T cell functions, and its efficacy depends on the recognition of tumor cells for destruction. Considering the factors at play, one could propose that anti-tumor responses will not occur if tumor cells are immunologically invisible to T cells. In this study, we tested a strategy based on the modulation of cancer cell's immunovisibility through HDAC inhibition. In a model (heterotopic and orthotopic) of mouse urothelial bladder cancer, we demonstrated that the use of intratumoral or intravesical HDACi in combination with systemic anti-PD-1 was effective at inducing curative responses with durable anti-tumor immunity capable of preventing tumor growth at a distal site. Mechanistically, we determined that protective responses were dependent on CD8 cells, but not NK cells. Of significance, in an *in vitro* human model, we found that fully activated T cells fail at killing bladder cancer cells unless tumor cells were pretreated with HDACi. Complementary to this observation, we found that HDACi cause gene deregulation, that results in the upregulation of genes responsible for mediating immunorecognition, *NKG2D* ligands and *HSP70*. Taken together, these data indicate that HDAC inhibition results in the elimination of the tumor cell's “invisibility cloak” that prevents T cells from recognizing and killing them. Finally, as checkpoint blockade therapy moves into the adjuvant setting, its combined use with locally administrated HDACi represents a new approach to be included in our current therapeutic treatment toolbox.

## Introduction

Bladder cancer is the sixth most common malignancy in the United States, and it is estimated that 81,190 patients received a new diagnosis of bladder cancer in 2018 (1). Approximately 80% of these patients will be found to have superficial bladder tumors at the time of diagnosis (2). Following transurethral resection, the gold standard adjuvant therapy for patients with intermediate and high risk non-muscle invasive bladder cancer is intravesical Bacillus-Calmette Guerin (BCG) (3). While BCG has been shown to reduce the risk of recurrence and delay disease progression, ~50% of patients will fail to respond to therapy (4–6). Further intravesical therapy options are limited in patients with BCG refractory and recurrent non-muscle invasive bladder cancer, and many of these patients will go on to require radical cystectomy or chemoradiotherapy (4–7). Therefore, the investigation into novel approaches in the management of high-grade, noninvasive bladder cancer is imperative.

Checkpoint blockade derives from the immune system's anti-tumor capabilities through the blocking of inhibitory signals that prevent the proper function of T cells (8). Currently, the use of monoclonal antibodies targeting cytotoxic T-lymphocyte-associated antigen-4 (CTLA-4), PD-1, and Program Cell Death Ligand 1 (PD-L1) has remained limited to unresectable, locally advanced and metastatic disease. The recent advent of checkpoint blockade immunotherapy in the treatment of urothelial cell carcinoma has provided new avenues for therapy in patients with urothelial cell carcinoma (9). Checkpoint blockade alone has provided a needed therapeutic venue for patients with advanced disease; however, clinical response occurs in only 20–30% of patients (10, 11).

On February 2020, the Food and Drug Administration (FDA) granted accelerated approval checkpoint blockade therapy for the treatment of some patients with for BCG-unresponsive, high-risk non-muscle invasive bladder cancer. However, clinical responses are expected to occur in only 20–30% of patients. It is possible that the anti-tumor effects of checkpoint blockade alone are limited if T cells are unable to recognize tumor cells as targets for destruction. In order to enhance clinical responses, we propose a therapeutic venue that could increase tumor recognition by the host immune system. Physiologically, cells must control the coiling and uncoiling of DNA around histones. This is accomplished in part by two families of enzymes, histone acetyl transferases (HAT), which promote transcription, and histone deacetylases (HDAC) which condense and transcriptionally silence chromatin. Thus, inhibition of HDAC results in an increase in acetylation of histone tails resulting in chromatin remodeling (12). While HDACi were first conceived as cytotoxic chemotherapeutic agents, further examination in several tumor models indicate that HDACi also enhanced tumor immunogenicity (13–22). These studies raised the possibility that HDACi could improve the efficacy of checkpoint inhibition through direct and indirect mechanisms. This hypothesis was tested in several clinical trials with encouraging results (15, 17, 19, 20, 23, 24). In this study, we evaluated the combined use of local HDACi (CI994 or SAHA) and systemic checkpoint blockade therapy (anti-PD-1 mAb) for the treatment of urothelial cell carcinoma of the bladder.

Studies have shown that certain HDAC family members are aberrantly expressed in urothelial bladder cancer (25–30). For example, studies have shown that high-grade tumors and high expression levels of HDAC1 are more likely to progress compared to all other patients (*p* < 0.05) (29). Based on these observations, we first sought to target HDAC1, hence the use CI994 (Tacedinaline), a selective inhibitor of HDAC1 with significant activity in a number of *in vivo* tumor models (31–33). Moreover, studies have shown high HDAC expression levels are found in 40–60% of all investigated urothelial carcinomas (HDAC-1: 40%, HDAC-2: 42%, HDAC-3: 59%) compared to normal urothelium (29). Based on this data, we also tested SAHA, a broad inhibitor of HDACs (class I and II HDACs) (34, 35). In our study, using models of mouse and human bladder cancer, we demonstrated that the combined use of local HDACi and systemic anti-PD-1 blockade was effective at inducing anti-tumor responses with durable anti-tumor immunity that was associated with the upregulation of genes responsible for mediating immunorecognition, *NKG2D* ligands and *HSP70*.

## Materials and Methods

### Bladder Cancer Cell Lines

Human bladder cancer cell line SW780 was purchased from ATCC. Mouse bladder cancer cell line MB49 was purchased from Sigma-Aldrich. MB49-luciferase (MB49-luc), was generated by first transfecting HekT cells with the F-luciferase plasmid using Lipofectamine 2000 (ThermoFisher). Supernatant from successful transfection (positive GFP signal under a fluorescent microscope) combined with polybrene was applied to wells plated with MB49 and centrifuged at 2,000 rpm for 2 h at 32°C. Transduced GFP-positive cells were cell-sorted, expanded and frozen in freezing media [heat-inactivated fetal bovine serum (Seradigm), 10% (DMSO)]. All bladder cancer cells were cultured in DMEM (Corning) supplemented with 10% heat-inactivated fetal bovine serum, 1% Penicillin/Streptomycin, and 2 mM L-glutamine (Corning).

### RNA Isolation and Microarray Analysis

To assess changes in gene expression, mRNA was extracted using mirVana mRNA Isolation Kit (Invitrogen) followed by cDNA conversion using SuperScript IV First-Strand Synthesis System at 500 ng/reaction (Invitrogen). Array-based gene expression analysis was performed using the NextSeq 550 System (Illumina).

### Anti-CD3-Activated Human T Cells

Healthy donor PBMCs (Key Biologics) were cultured with 100 ng ml-1 anti-CD3 (clone OKT3) for 5 days with IL-2 (300 IU ml-1) and IL-15 (100 ng ml-1). T cell enrichment and activation were corroborated by flow cytometry.

### *In vitro* T Cell Killing Assay

SW780 cells were incubated with or without SAHA at 5 μM. After 12 h, cells were extensively washed to remove traces of HDACi. Treated SW780 cells were incubated with or without OKT3-activated human T cells at a 5:1 (Effector: Target) ratio. Following 24 h, wells were washed, and floating cells removed. Remaining bound cancer cells were stained with DAPI. Each well was photographed under a fluorescence microscope for nuclear staining, DAPI+ cells. The enumeration of the remaining cells per well was conducted by using a computer-based automatic counting algorithm (Image J, NIH).

### Mice

Animal experiments were conducted in accordance with Loyola University Chicago Institutional Animal Care and Use Committee guidelines. Six to eight week-old C57BL/6 male and female mice were purchased from The Jackson Laboratory. All mice were housed in a specific-pathogen-free facility at Loyola University Chicago, Cardinal Bernardin Cancer Center.

### Intradermal Mouse Tumor Model

Tumor cells were implanted through flank intradermal injection of 2 × 10^5^ MB49 cells. Mice bearing tumors of 0.5 cm in any direction were treated i.p. with 200 μg IgG, 200 μg anti-PD-1 (BioXcell) once per week, intratumoral SAHA (50 of 10 μM SAHA), or combination (SAHA+anti-PD-1). Control mice were intratumorally injected with 50 μL DMSO-PBS. CD8 and NK cell depletions were conducted by i.p injection of 250 μg Clone 2.43 or 250 μg anti-Asialo GM1 antibody (36). Depletion was confirmed by flow cytometry in sentinel mice. Control groups received hamster IgG. To assess for long-term tumor immunity, mice that rejected tumors were rested for an additional 30 days and received a second MB49 tumor challenge in the contralateral flank alongside a control group.

### Intravesical Mouse Tumor Model and Intravesical Tumor Treatment

Tumor implantation was conducted as previously described (37). Briefly, Female B6 mice were intravesically catheterized via a 24G catheter while under constant 3% isoflurane gas anesthesia. After bladder emptying, 80 microliters of 0.125% trypsin in DMEM base medium were instilled in the bladder. After 15 min, trypsin was removed and 50 microliters of PBS containing 2 × 10^5^ MB49-Luc cells were intravesically instilled for 50 min. Intravesical and systemic treatments were conducted in anesthetized mice once tumor take has been confirmed by bioluminescence (~3–5 days after implantation). Briefly, tumor-bearing mice were separated in groups, emptied bladder and simultaneously treated for 45 min with PBS-DMSO (control) or CI-994 in DMSO in 50 microliters. Tumor growth was followed every 5 days by *in vivo* bioluminescent imaging, IVIS Spectrum *in vivo* Imaging System (Perkin Elmer). Control mice received 200 μg IgG or 200 μg anti-PD-1, i.p. once per week. Mice were only treated once.

### Histopathologic Assessment of Bladder Integrity Following Treatment

Treated mice underwent surgical removal of the urinary bladder. The bladders were subsequently sectioned in the midsagittal plane and embedded in paraffin. Sections of 5-μm were obtained from tissue blocks and stained with hematoxylin and eosin (H&E). Cell infiltration and the presence of residual tumors were assessed.

#### Flow Cytometry

Mouse bladders bearing MB49 tumors were surgically excised followed by mechanical and enzymatic (Liberase, Sigma) dissociation. Fluorochrome-conjugated antibodies against Fc-receptor, CD3, NK1.1, CD4, CD8, CD11c, CD19, F4-80 and Gr-1 (eBioscience) were used. Stained cells were analyzed by flow cytometry on LSR Fortessa (BD Biosciences). For all the flow cytometry data analysis, dead cells were excluded from the analysis by using Zombie Aqua viability dye (BioLegend).

### Statistical Analysis

Differences in gene expression were determined by ANOVA analysis. Statistical differences in flow cytometric analysis were determined by Student two-tailed *t-*test. Kaplan-Meier curves were generated using GraphPad Prism 8 to detect differences in tumor regression and tumor-free survival.

## Results

### Local HDACi Treatment in Combination With Systemic Anti-PD-1 Induces Immune-Mediated Tumor Regression and Durable Tumor Immunity

**Figure 1:**
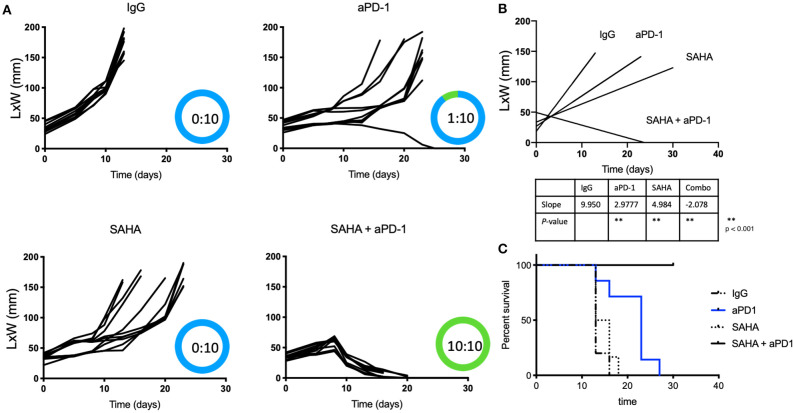
Intratumoral HDACi enhances systemic anti-PD-1 anti-tumor immunity. **(A)** MB49-bearing male B6 mice (10 per group) with tumors of 0.5 cm, were treated as follows: IgG control, systemic anti-PD-1, intratumoral SAHA, combination SAHA and anti-PD-1. Tumor growth was depicted as LxW in mm and plotted against time in days. The number of mice presenting tumor are represented in blue and tumor-free mice in green. Tumor free vs. total mice are indicated inside the circle. **(B)** Tumor growth slopes (L × W mm) are shown comparing the different groups, **p < 0.001. **(C)** Kaplan-Meier curves for each treated group are shown. Survival is defined as the tumor reaches 1.5 cm in any direction. Mice were monitored for 30 days. These data are representative of two independent experiments.

Because of HDACi's role in increasing tumor immunogenicity, we tested if the use of local (intratumoral) HDACi could enhance the systemic effects of anti-PD-1 therapy. Only male mice were used as immune responses against the Y antigen by female mice has been reported in the MB49 model (38). Mice bearing 0.5 cm intradermal tumors received (i.p.) IgG, anti-PD-1, intratumoral SAHA (or DMSO/PBS), or combination anti-PD-1 (systemic)/SAHA (intratumoral) Figures 1A–C show that all mice that received control antibody underwent rapid tumor growth. Significant alterations in the tumor growth slope were observed in mice treated with anti-PD-1 antibody or intratumoral SAHA alone (*p* < 0.05); however, no tumor regression was observed. Of the mice that received systemic anti-PD-1 antibody alone, only one developed an effective anti-tumor response. Of the group treated with SAHA alone, 10 of 10 mice developed tumors without regression. In contrast, 10 of 10 mice that received a combination of intratumoral SAHA and systemic anti-PD-1 therapy had complete regression of their tumors and 100% survival (*p* < 0.05).

### Anti-Tumor Immunity After Combined HDACi Treatment With Systemic Anti-PD-1 Is Dependent on CD8 T Cells, but Independent of NK Cells

**Figure 2:**
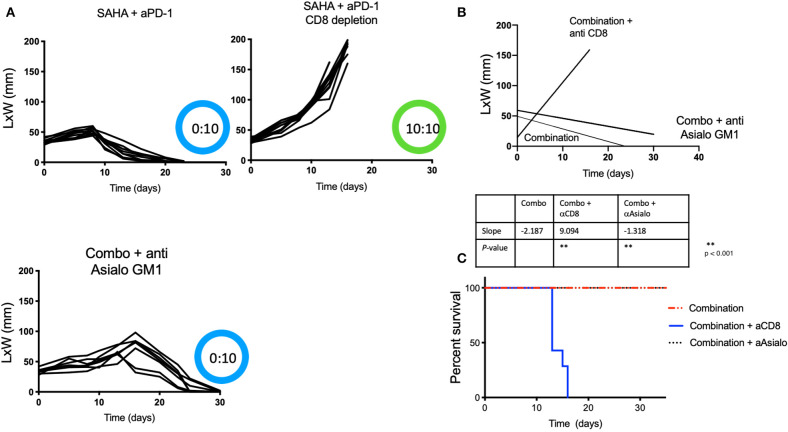
Protective anti-tumor responses by HDACi and PD-1 are CD8-mediated and NK independent. Tumor-bearing mice treated as Figure 1, also received depleting anti-Asialo GM1 or anti-CD8. **(A)** Tumor growth was depicted as L × W in mm and plotted against time in days. The number of mice presenting tumor are represented in blue and in tumor-free mice in green. **(B)** Tumor growth slopes (L × W) are shown comparing the different groups, **p < 0.001. **(C)** Kaplan-Meier curves for each group are shown. Survival is defined as the tumor reaches 1.5 cm in any direction. Mice were monitored for 30 days. These data are representative of two independent experiments. The number of mice presenting tumor are represented in blue and tumor-free mice in green. Tumor free vs. tumor-bearing mice are indicated inside the circle.

We next sought to determine if the anti-tumor immune responses observed following treatment with the combination of HDACi and anti-PD-1 antibody were mediated by CD8 T cells or NK cells. Tumor-bearing male mice receiving intratumoral SAHA and systemic anti-PD-1 were also treated with CD8 or anti-Asialo GM1 antibody depleting mAbs. We found that none of the ten mice that had undergone CD8-immune depletion derived benefit from combined therapy. All mice that were CD8 depleted developed tumors with a slope that was indistinguishable from the control group (*p* > 0.05). In contrast, all mice that were NK depleted maintained strong anti-tumor immunity (Figures 2A–C) (*p* < 0.05). NK depletion was confirmed by flow cytometry (Supplementary Figure 1).

### Intratumoral HDACi and Systemic Anti-PD-1 Induce Durable Immunity Against a Distal Tumor

**Figure 3:**
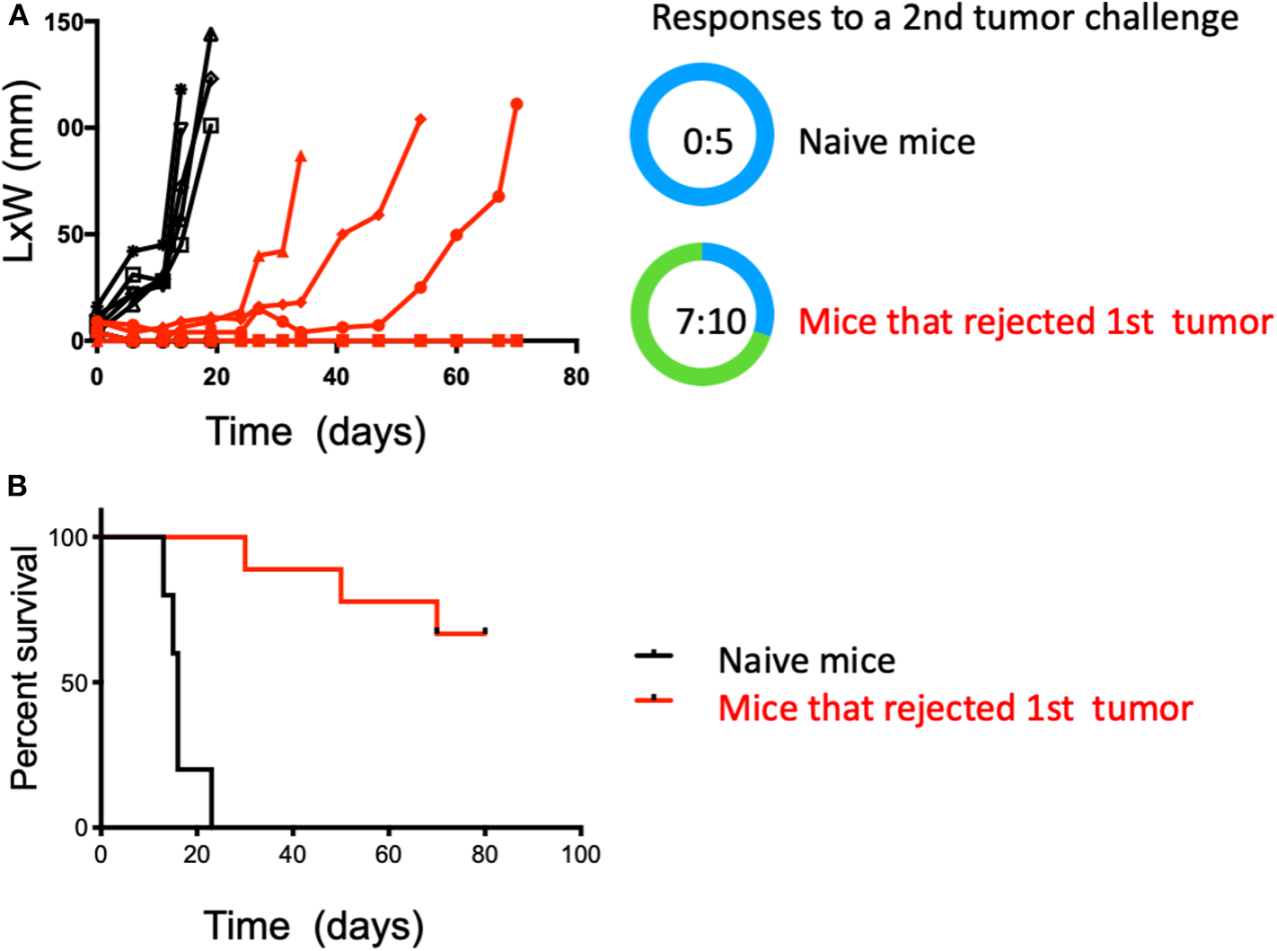
Combined treatment with systemic anti-PD-1 and intratumoral HDACi results in durable anti-tumor immunity that protects against a second tumor challenge. Surviving mice treated with HDACi and anti-PD-1 mAb were rested for 30 days and challenged in the opposite flank with MB49 cells. Non-treated group = 5 mice; treated mice that rejected the first MB49 tumor challenge = 10 mice. **(A)** Five of five non-treated mice developed tumors. In the group of mice that rejected the first tumor (10:10), only three developed secondary tumors. **(B)** Kaplan-Meier curves, surviving mice were monitored for 80 days. Survival is defined as tumor reaching 1.5 cm in any direction. The number of mice presenting tumors are represented in blue and tumor-free mice in green. Tumor free vs. total mice are indicated inside the circle.

To test if the observed anti-tumor immunity was durable and capable of affecting a tumor at a distal site, mice from the combination therapy arm that had undergone complete tumor regression were challenged again, but in the contralateral flank with intradermal MB49 cells after 30 days of resting. These mice received no further therapy after the second inoculation, therefore, changes in tumor growth could be only be attributed to the previously generated immunity. A group of naïve and untreated mice was also challenged to establish MB49 cells *de novo* tumor growth. Not surprisingly, all mice (5 of 5) in the control group rapidly developed tumors. In contrast, seven of 10 in the previously treated mice (HDACi + anti-PD-1 mAb) rejected a second tumor challenge and remained tumor-free 3 months after initial tumor inoculation (*p* < 0.05) (Figures 3A,B).

### Intravesical HDACi in Combination With Systemic Anti-PD-1 Induces Tumor Regression

**Figure 4:**
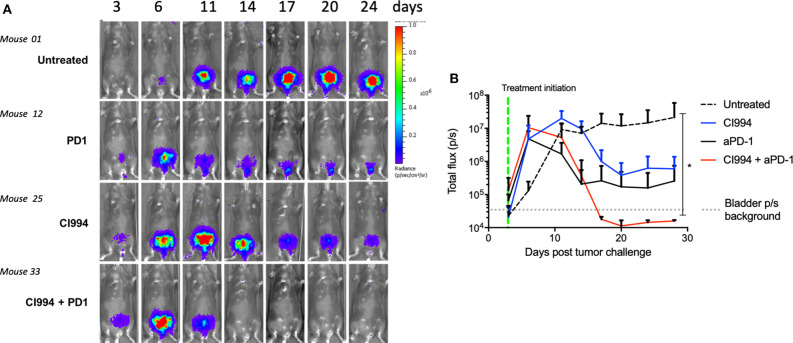
*In vivo* bladder cancer regression following combined intravesical HDACi treatment and systemic T cell immune activation by PD-1 blockade. Once tumor take was confirmed by *in vivo* imaging (day 4), MB49-bearing female B6 mice were treated. Mice were randomized in groups: untreated, intravesical CI-994, systemic anti-PD-1, combination CI-994 and anti-PD-1. Mice were monitored for at least 30 days. These data are representative of two independent experiments. **(A)** *In vivo* imaging examples of intravesical bladder cancer tumor progression in individual mice. **(B)** Graphical representation of intravesical tumor growth (total flux in photons per second) in treated mice. Ten mice per group were used, data are representative of two independent experiments. *p < 0.05.

To assess the feasibility of intravesical delivery of HDACi for the treatment of bladder cancer, we used an orthotopic bladder cancer model, MB49-luc. In this case, we used female mice given the possibility of catheterizing the urethra. We confirmed intravesical tumor take by *in vivo* bioluminescent visualization (Figure 4A). Tumor take was typically observed at day 3–4 after intravesical instillation. As observed in the intradermal model, 10 of 10 female mice treated with the combination of intravesical HDACi (CI-994) and systemic PD-1 blockade developed curative anti-tumor responses in their bladders (*p* < 0.05). Ten of 10 mice that received sham treatment developed tumors. Mice that were treated with CI-994 or anti-PD-1 antibody alone, although generated a tumor size reduction, failed at achieving curative tumor regression (Figure 4B). A comparison between the combination treatment and monotherapies indicates no statistically significant differences (*p* > 0.005). MB49-luc maintained luciferase expression for the duration of the *in vivo* experiment (>30 days), as shown by luminescence in tumor-bearing mice that received no treatment. The presence or absence of tumor tissue was corroborated by macro and microscopic analysis of the bladder.

### HDAC Inhibitor in Combination With Anti-PD-1 Induce Immune Infiltration and Tumor Destruction *in situ*

**Figure 5:**
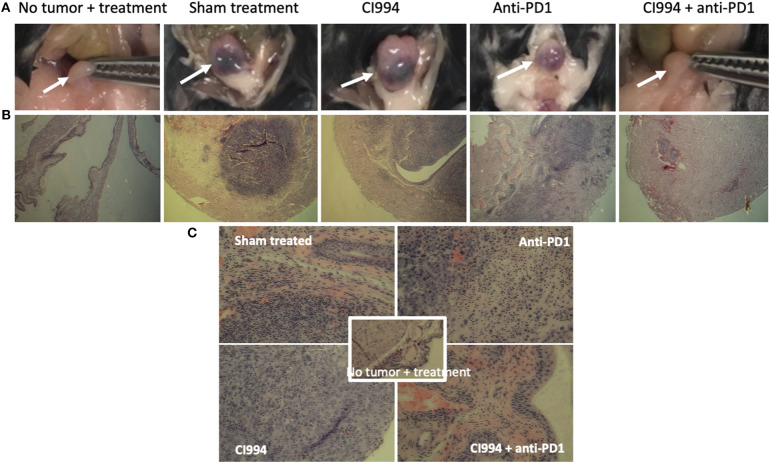
Intravesical HDACi in combination with systemic anti-PD-1 induce immune infiltration and tumor destruction *in situ*. Bladders from treated mice were surgically resected and prepared for H&E staining and histological analysis after tumors were not present by *in vivo* imaging (~d12). Groups: mice were not tumor challenged but received combined treatment; tumor-bearing mice received sham treatment; tumor-bearing mice received intravesical CI-994 or anti-PD-1 or their combination. **(A)** Bladder macroscopic view are shown of representative mice. **(B)** Bladders of mice treated intravesically with CI-994 show the presence of tumor cells. **(C)** Anti-PD-1 alone induces immune infiltration, but tumor cells were also present. Bladder of mice treated intravesically with HDACi (CI-994) and systemic PD-1 antibody combination show immune infiltrate with substantial or complete tumor cell clearance. Combination treatment caused no apparent changes in the bladder integrity.

**Figure 6:**
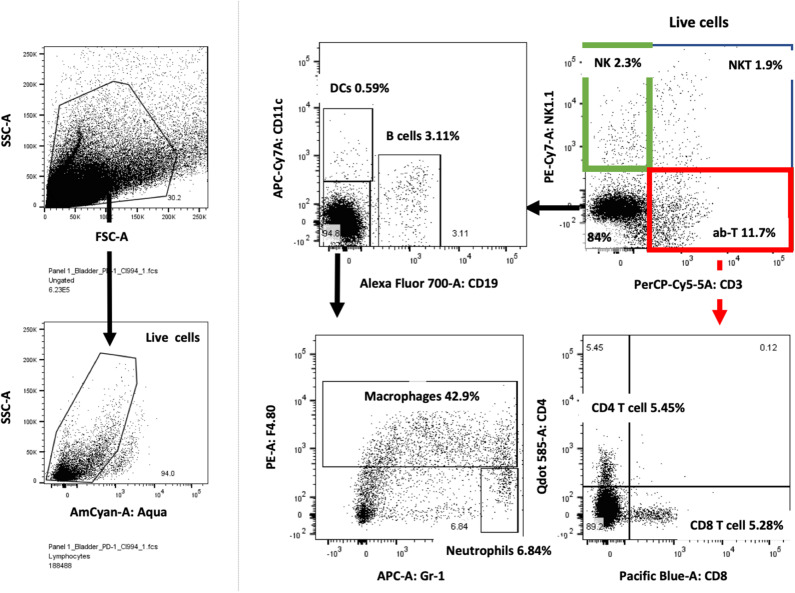
Bladder cancer gating strategy for flow cytometry analysis. Tumor-bearing bladders from treated mice were surgically resected at day after treatment, dissociated and prepared for flow cytometry analysis. Live lymphocytes were analyzed for T cells subdivided into CD4 and CD8 cells, NK, NKT, DCs, macrophages, neutrophils and B cells. Example of one tumor-bearing bladder is shown.

Following the completion of intravesical therapy, the bladders of these mice were macroscopically visualized (Figure 5A) and surgically removed for histopathologic analysis (H&E) (Figures 5B,C). Untreated bladders showed extensive invasive carcinoma. Mouse bladders treated with intravesical CI-994 demonstrated a mild or no reduction in invasive carcinoma. Those treated with anti-PD-1 alone showed immune infiltration, but prominent invasive carcinoma remained. Mouse bladders that had received combination therapy demonstrated immune infiltration with minimal or no residual carcinoma. Next, we sought to determine if intravesical HDACi exposure and systemic PD-1 blockade treatment result in alteration in bladder integrity that could preclude their clinical use. We microscopically analyzed (H&E) the bladders of treated mice that received combination therapy. Histopathology analysis revealed no deleterious changes in the integrity of bladders of mice treated with CI-994 and PD-1 blockade compared with mice receiving no treatment (Figure 5C, center). To gain further information regarding the cellular composition among the different treatment groups, we analyzed the bladder of tumor-bearing mice 6 days after treatment by flow cytometry. We determined the frequency of T cells, B, macrophages, dendritic cells (DCs) and neutrophils in bladder tumor of mice treated with HDACi, anti-PD-1, combination or no treatment. In Figure 6 and Table 1, we show that treatment with HDACi alone was insufficient to cause significant changes in the immune composition of the bladder. In contrast, mice treated with aPD-1 mAb, the frequency of CD3 and CD8 T cells was higher than in untreated or HDACi-treated mice (*P* < 0.005). These effects of a-PD1 mAb were also observed in the group of mice treated with both aPD-1 and HDACi (*P* < 0.05).

**Table 1:** Frequency of immune cells in tumor-bearing bladders.

	**CD3+CD8+**		**CD3+CD4+**		**B cells**		**DCs**		**Macrophages**		**Neutrophils**		**NK**		**NKT**	
	**Mean**	**SD**	**Mean**	**SD**	**Mean**	**SD**	**Mean**	**SD**	**Mean**	**SD**	**Mean**	**SD**	**Mean**	**SD**	**Mean**	**SD**
Untreated	1.847	0.8701	4.493	0.8064	4.097	0.8173	0.5567	0.2173	50.6	7.374	5.103	0.7823	2.217	0.08	1.805	0.755
f1994	4.737	2.665	5.69	1.114	5.35	1.074	0.5833	0.0702	44.57	5.404	8.6^*^	0.7119	1.983	0.98	2.123	0.989
aPD-1	6.09^*^	1.472	8.757^*^	1.862	7.15	2.405	0.78	0.0866	43.13	3.758	11.7^*^	3.671	2.331	1.44	2.353	1.1124
aPD-1+Cl994	5.287^*^	1.04	6.437	1.752	6.697	3.116	0.96	0.3804	47.47	4.994	8.753	2.296	2.447	0.89	2.024	1.07

*Bladders from treated mice were surgically resected, dissociated and prepared for flow cytometry analysis as shown in Figure 6. The frequency (the % positive cells of a specific phenotype among 10,000 events) of CD3, CD4, CD8, NK, NK T, B cells, DCs, macrophages, and neutrophils are shown. Statistical significance was calculated by student t-test*,

* *P < 0.05. Bold indicates statistical significance*.

### Exposure to HDACi Results in Increased Recognition and T Cell-Mediated Tumor Cell Killing in Human Bladder Cancer Cells

**Figure 7:**
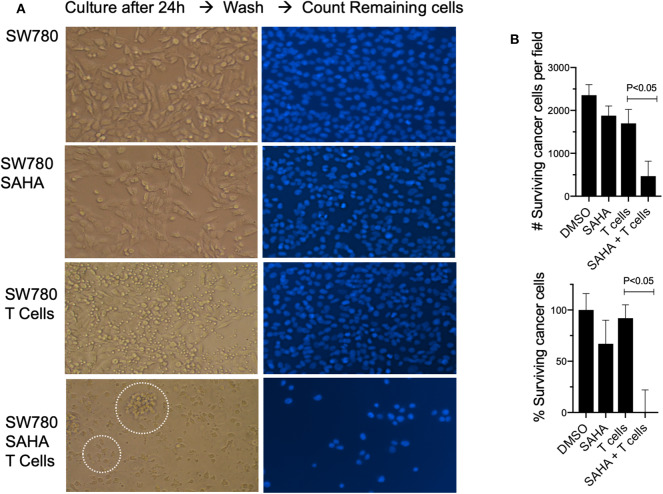
Pretreatment with SAHA enhances T cell-mediated cytotoxicity in human bladder cancer cells. **(A)** SW780 human bladder cancer cells were treated with either DMSO or SAHA and activated T cells were then added to culture (left). T cells were then washed, and remaining cells were stained for DAPI (right). Dotted circles indicate T cells and T cells swarming around a tumor cell. **(B)** Remaining cells following treatment were also counted to determine viability following treatment.

To establish relevance in the human setting, we next examined if exposure of a human bladder cancer cell line SW780 to HDACi (SAHA) would impact their recognition and killing by fully activated human T cells. To do this, T cells and SW780 were both HLA-A2.1 matched. Exposure of SW780 to SAHA alone resulted in a modest cytotoxic effect with a 22% reduction in viable tumor cells compared to control-treated cells (*p* < 0.05). Incubation of SW780 (DMSO treated) with activated T cells alone showed some direct T cell-mediated cytotoxicity with a 30.4% reduction in tumor cells when compared to SW780 cells alone (*p* < 0.05). Notably, tumor cell pretreatment with SAHA augmented activated T cell-mediated cytotoxicity with a 73.9% reduction in viable tumor cells compared to cells treated with DMSO and T cells (*p* < 0.05) (Figures 7A,B). Visual analysis of the combined shows fully activated T cells failing to engage tumor cells (SW780 + T cells group), Figure 7A. Notably, in the SW780 SAHA treated + T cells group, T cells are clearly seen swarming around tumor cells.

### HDACi Causes Gene Deregulation in Human Bladder Cancer Cells

**Figure 8:**
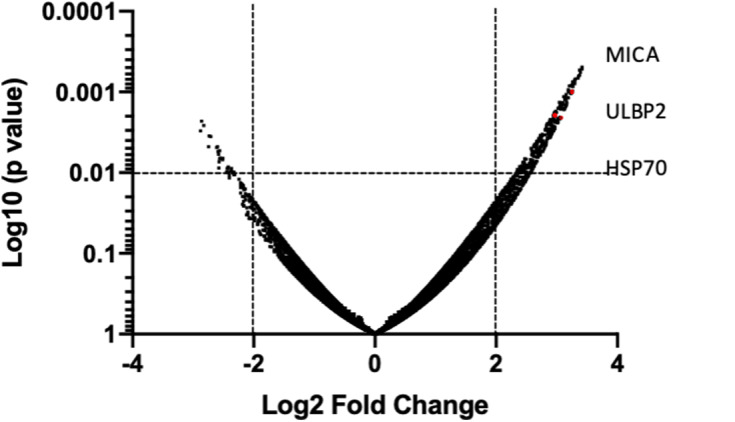
HDACi induces gene expression changes. Global gene array comparing human SW780 cells treated with SAHA vs. DMSO. Volcano plots of genes were created by plotting Log2 fold change in relationship with Log10 (*p*-value). Significance was considered if *p* < 0.01 was shown. Genes in the upper and left quadrant changed ±2 folds and *p* < 0.01.

In order to provide a tentative explanation to the effects observed and perhaps a potential intersection with T cell-mediated anti-tumor pathways, we examined the capacity of HDACi to alter gene expression in human bladder cancer. *In vitro* exposure of the human bladder cancer cell line, SW780 to HDACi (SAHA) results in the alteration of gene expression compared with DMSO-treated cells (Figure 8). Baseline gene expression was normalized to 0. Sixty-nine genes were found to have a 2-fold increase or greater, and 19 genes were found to have a 2-fold decrease or greater (*p* < 0.05), (Supplementary Table 1). Notably, an in-depth analysis indicates that among the group of genes that were upregulated, the immunologically relevant genes encoding ligands for NKG2D, MICA and ULBP2 and the heat shock protein 70 (HSP70) were among those upregulated.

## Discussion

Using a mouse and human model of bladder cancer, we demonstrated that the combined use of local HDACi and systemic anti-PD-1 was effective at inducing curative CD8 T cell immune responses against primary lesions with durable anti-tumor immunity against a secondary and distal tumor. We also demonstrated the relative safety and applicability of this strategy in an orthotopic intravesical bladder tumor model. We show that inhibition of HDAC in a human bladder cancer cell line facilitates their recognition and killing by T cells. Moreover, we show that HDACi causes tumor cell gene deregulation, characterized by upregulated genes responsible for mediating cellular stress, as shown by the increased expression of *NKG2D* ligands and *HSP70*.

HDAC inhibitors were first conceived with the idea of using them as chemotherapeutic/cytotoxic agents. Indeed, some of them, Romidepsin (Istodax; Celgene) and Belinostat (Beleodaq; Spectrum Pharmaceuticals) are FDA approved for the treatment of cutaneous T cell lymphoma (CTCL) and peripheral T cell lymphoma (PTCL) (39), and multiple myeloma (40, 41). HDACi act by affecting the DNA repair machinery, altering gene expression leading to post-translational modifications to proteins HDACi have been shown to stop the proliferation of cancer cells, stimulate apoptosis and induce cell cycle arrest. Moreover, inhibition of HDACs in tumor cells offers additional potential benefits, namely augmentation of cancer immunogenicity (13–22).

Studies have shown that certain HDACi can increase the expression of tumor-shared antigens and the expression of MHC class I and class II in melanoma cells (42, 43), or expression of HSP70 and HSP90 on various hematopoietic cancer cells (13, 44). In a panel of NSCLC cell lines *in vitro*, mocetinostat (inhibitor of class I/IV histone deacetylases) upregulated PD-L1 and antigen presentation genes including class I and II human leukocyte antigen (HLA) family members. In a syngeneic tumor model, mocetinostat decreased intratumoral regulatory T cells (Tregs) and potentially myeloid-derived suppressor cell (MDSC) populations and increased intratumoral CD8 T cell populations (20).

Studies have shown that co-treatment with HDACi and checkpoint inhibitors improved treatment outcomes against colorectal carcinoma, CT-26 and breast cancer 4T1 tumors (16). In these studies, protective responses were associated with the depletion of myeloid-derived suppressor cells (MDSCs). More recently, a study using a mouse model of hepatocellular carcinoma showed that systemic HDACi (Belinostat) combined with the simultaneous blockade of CTLA-4 led to the decrease of regulatory T cells and complete tumor rejection (22). Recently, a study showed that HDAC6 inhibitors improve anti-PD-1 immune checkpoint blockade therapy by decreasing the anti-inflammatory phenotype of macrophages and down-regulation of immunosuppressive proteins in tumor cells (21). A study using prostate (LNCAP) and breast (MDA-MB-231) carcinoma cells demonstrates that treatment with either the pan-HDAC inhibitor vorinostat or the class I HDAC inhibitor entinostat results in T-cell mediated lysis *in vitro*. Moreover, the authors show that part of the mechanisms responsible for the recognition of HDACi-treated cells is mediated by the ER stress-responsive element (45). Additional studies have shown that the effects of HDACi can extend to changes in the antigen processing machinery. Using melanoma B16 cells, the authors demonstrate that the HDACi, TSA increases the expression of several components of the antigen processing machinery, including TAP-1, TAP-2, LMP-2, and Tapasin (46, 47). Importantly, HDACi have also been shown to increase the expression of costimulatory molecules on the surface of tumor cells, such as CD40, CD80, and 4–1BB. For example, the HDACi, panobinostat, has been shown to enhance immune recognition of melanoma by immune cells. In this study, the effects were correlated to the upregulation of costimulatory molecules CD40 and CD80 (43). Moreover, a study using leukemia cells found that HDAC inhibition results in the upregulation of 4-1BBL/TNFSF9 (48). In our study, we found that, indeed, inhibition of HDACs in bladder tumor cells results in their recognition and killing by CD8 T cells. Giving a possible explanation for the mechanisms, our study suggests that HDACi induces cellular stress in bladder cancer cells, as shown by the increased expression of *NKG2D* ligands and *HSP70*.

Physiologically, NKG2D ligands serve as signals to the immune system of catastrophic cell damage and the need for immune-mediated destruction. This is consistent with prior work from separate studies by L. Lanier and D. Raulet groups where genetically-driven expression of NKG2D ligands in tumors was sufficient to cause tumor rejection (49, 50). Given the canonical function of NKG2D, which is to recognize cells undergoing genotoxic stress response, one can speculate that HDAC inhibitor-treated tumor cells expressing NKG2D ligands would be visible to cytolytic cells for destruction, namely by NK and CD8 T cells. Consistent with this possibility, a study in myeloma cells shows that valproic acid (VPA) upregulates both protein and mRNA expression of NKG2D ligands (MICA/B) and ULBP2 (51). Based on these studies, the increase of NKG2D ligands suggests that HDAC inhibitor-treated cells triggers tumor cell destruction through a suicide by proxy mechanism. Our data support this assumption, as only HDAC inhibitor-treated tumor cells are recognized and killed by CD8 T cells. Interestingly, our data also indicate that HDAC inhibition modulates the expression of HSP70 and ligands for NKG2D in bladder cancer cells. This dual effect is mechanistically consistent, as the expression of both genes is controlled by the heat shock factor 1 (HSF1). This is a transcriptional factor that activates the transcription of genes coding for proteins that protect the cell against harmful stresses (52). Based on studies on the role of HSPs in the cell stress response (13), we interpret the increase in levels of HSP70 as a cellular attempt to survive. However, the function of HSP70 also extends to their participation as a peptide-chaperone to be taken by professional antigen-presenting cells (APC), leading to T cell priming (53). Studies have shown that immunization with a lymphocytic choriomeningitis virus peptide mixed with HSP70 results in protective antiviral immunity and antigen-reactive CD8 T cells (54). Similar results have been shown that vaccination with HSP preparations elicits a CD8 T cell responses *in vivo* (55). Based on these studies and our observations, one can propose that HDACi-related immunity is first mediated by the recognition of NKG2D ligands on the surface of treated tumor cells, and then followed by HSP70-mediated priming of T cells. Moreover, our groups recently demonstrated that NKG2D signaling in CD8 T cells at the time of the effector killing phase results in the acquisition of a transcriptional program that poised T cells with the potential to become long term memory T cells, we termed this process memory certification (56).

We show that the HDACi treated human bladder cancer cell line (SW780) can be recognized by human T cells. In this experiment, it is important to note that while the tumor and T cells were HLA-A2.1 matched, other histocompatibility proteins are expected to be different, leading to some level of allo-responses. However, our data demonstrate that even under these conditions, cancer cells are poorly recognized and destroyed by T cells. Our data clearly show that in contrast with the results observed in HDACi treated cancer cells, killing by T cells was negligible in non-HDACi treated cells. These data indicate that modulation of HDAC in tumor cells is sufficient to render them visible and susceptible to T cells. While our *in vitro* experiments demonstrate that HDACi have similar effects on bladder cancer cell lines, this has yet to be shown in highly heterogeneous tumors, which comprise the vast majority of *in situ* human bladder tumors. As in most preclinical models, a mouse cancer cell line may not completely reflect human bladder cancer patients. However, we believe the benefits of this model are novel as well as practical.

In our therapy experiments, some mice failed to develop curative responses upon combined therapy. However, anti-tumor responses were not absent, the tumor growth curves were significantly different to non-treated mice, tumors appeared later and grew slower. These data indicate the existence of anti-tumor response, however, insufficient to mediate tumor rejection.

However, the mechanisms mediating the tempering of the anti-tumor response could be multiple. It is possible that in this mice, inadequate number of anti-tumor T cells were generated or that immune suppressive mechanisms (i.e., TGF-b or Tregs) dominated the response (57, 58). In any case, it would be uncommon to see any therapy that provides 100% curative responses. We provide non-tangential data demonstrating that CD8 T cells mediate the anti-tumor response. We show that depletion of CD8 T cells results in the complete abrogation of anti-tumor responses upon combined treatment. These data indicate that the cellular mediators of tumor rejection upon combined treatment are CD8 T cells and that responses depend of the combined use of HDACi and anti-PD-1 mAb.

We observed differences in immune infiltration overserved during the flow cytometry and the histologic analysis. This can be explained by the differences in the time points when the samples where studied. The flow cytometry analysis of tumor-bearing bladders was conducted 3 days after the initiation of treatment. What is seen in the flow cytometry represents the active response to treatment. The histology was conducted 12 days after treatment, once tumors have disappeared in the combination group. The response seen in the histology represents the overall outcome of the treatment (after tumor were no longer detectable by *in vivo* imaging). Under this perspective, the data presented are consistent with the expansion phase, followed by the tumor clearance and contraction phase.

Analysis of the flow cytometry pattern in the bladder shows a population of T cells that are express CD3 but not CD8 or CD4 markers (DN). Studies have shown that these T cells are found in the thymus. In the peripheral, DN T cells have been shown to be involved in immune regulation and tolerance, as well as in host defense and inflammation. While we do not know the function of these population in the observed responses, current literature shows dual effects, inflammatory and suppressor. For example, DN cells have been shown to prevent allograft rejection, graft-vs.-host disease, and autoimmune diabetes (59). In contrast, studies have shown that DN T cells can protect against infections (60, 61). It is plausible that HDACi may also affect the DN cell population, by making less suppressive or more pro-inflammatory. Given these observations and their high frequency in tumor-bearing bladders, we believe that this population merits further studies.

While HDACi are currently available for systemic use in other cancer types, toxicity remains an issue (62, 63). The use of intravesical delivery of this agent may avoid the risks of systemic toxicity while allowing for local delivery directly to target tissue. Furthermore, this reduces the possibility of compounding adverse immune-related events with the administration of both therapeutic agents. It should also be noted that the anti-tumor effects of combination therapy are not only effective in inducing tumor regression but are also long-lasting. This provides a potential advantage in a malignancy with high rates of local recurrence.

The data presented in this study support the hypothesis that HDAC modulation in cancer cells can remove the tumor cells “invisibility cloak” that prevents T cells from recognizing and killing them. Furthermore, these data support a therapeutic route that could increase tumor recognition by the host immune system. Our preclinical investigation of the use of local HDACi in combination with systemic anti-PD-1 provides clinical investigators with an applicable approach that mediates anti-tumor activity with durable clinical response while mitigating the risk of side effects. This novel therapeutic treatment may provide new opportunities for patients with localized bladder cancer and should be further explored in the context of a clinical trial.

## Data Availability Statement

The raw data supporting the conclusions of this article will be made available by the authors, without undue reservation, to any qualified researcher.

## Ethics Statement

The animal study was reviewed and approved by Loyola University Chicago IACUC.

## Author Contributions

JG-P: study concept and design. BB, CE, CP, AB, SH, LP-R, KP, and MD: experiments, analysis, and interpretation of data. BB: drafting of the manuscript. CV-J, EH, GG, and JG-P: critical revision of the manuscript for important intellectual content.

## Conflict of Interest

The authors declare that the research was conducted in the absence of any commercial or financial relationships that could be construed as a potential conflict of interest.

## Supplementary Material

Supplementary Figure 1 Anti-Asialo GM1 antibody depletes NK cells *in vivo*. **(A)** Dot plot of spleens from sentinel mice that were treated with anti-Asialo GMI antibody. Cells were analyzed for CD3 and NK1.1 marker expression 3 days later by flow cytometry. **(B)** Representative plot of eight mice. ^*^^*^^*^^*^*p* < 0.0001.

Supplementary Table 1 Gene expression changes by HDAC inhibitor on the human bladder cancer cell line (SW780). Baseline gene expression was normalized to 0. Sixty-nine genes were found to have a 2-fold increase or greater, and 19 genes were found to have a 2-fold decrease or greater (*p* < 0.05).
