# Supplementary figures and images for: Inhibition of Histone Deacetylase (HDAC) Enhances Checkpoint Blockade Efficacy by Rendering Bladder Cancer Cells Visible for T Cell-Mediated Destruction
Source: Front Oncol. 2020 May 15;10:699. doi: 10.3389/fonc.2020.00699 (PMC7243798; doi:10.3389/fonc.2020.00699)

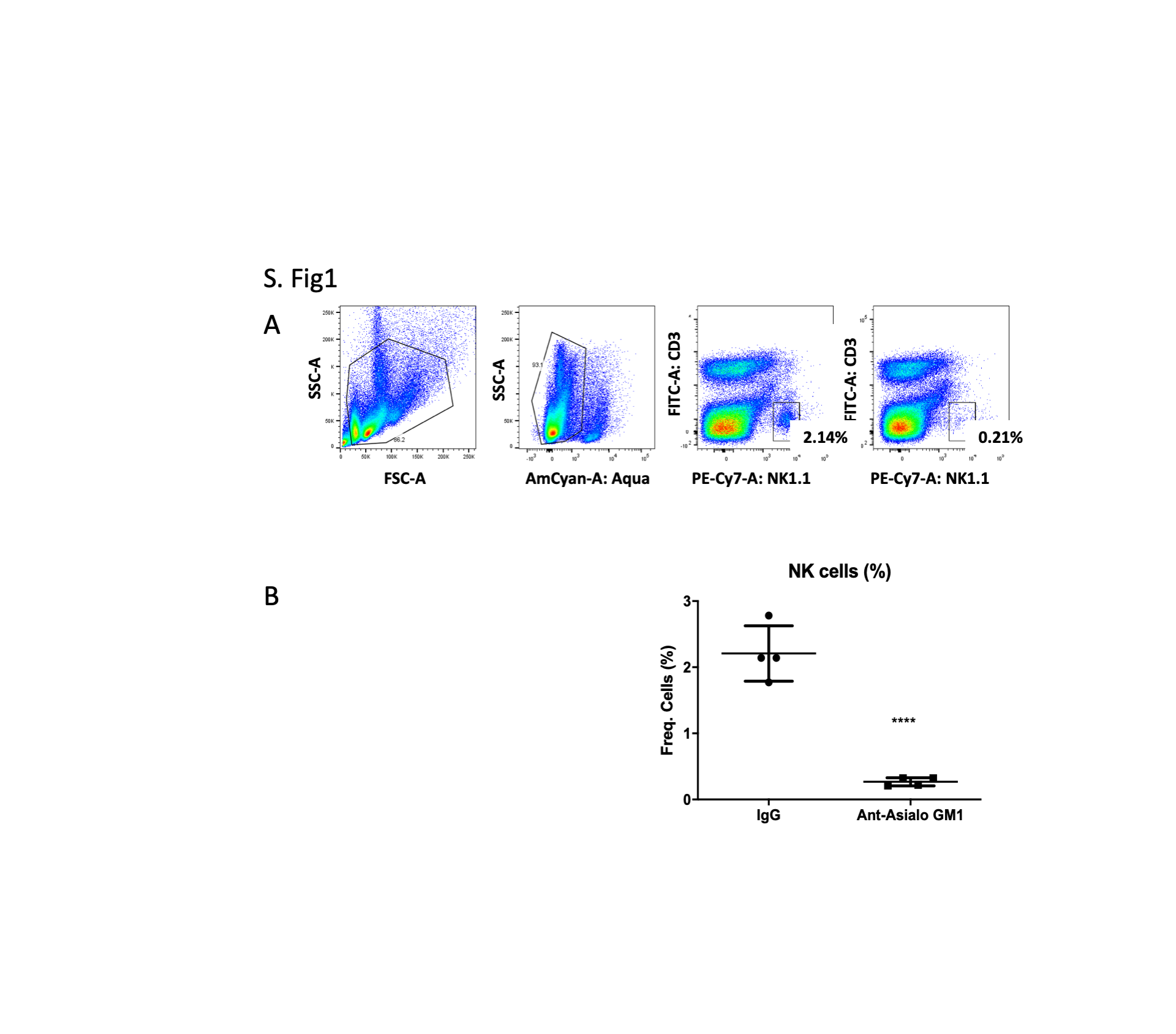

Supplement: Supplementary Figure 1 — Anti-Asialo GM1 antibody depletes NK cells in vivo. (A) Dot plot of spleens from sentinel mice that were treated with anti-Asialo GMI antibody. Cells were analyzed for CD3 and NK1.1 marker expression 3 days later by flow cytometry. (B) Representative plot of eight mice. ****p < 0.0001. [file Image_1.tiff]
