# Supplementary material for: Inhibition of Histone Deacetylase (HDAC) Enhances Checkpoint Blockade Efficacy by Rendering Bladder Cancer Cells Visible for T Cell-Mediated Destruction
Source: Front Oncol. 2020 May 15;10:699. doi: 10.3389/fonc.2020.00699 (PMC7243798; doi:10.3389/fonc.2020.00699)
Supplement: Supplementary Table 1 — Gene expression changes by HDAC inhibitor on the human bladder cancer cell line (SW780). Baseline gene expression was normalized to 0. Sixty-nine genes were found to have a 2-fold increase or greater, and 19 genes were found to have a 2-fold decrease or greater (p < 0.05). [file Data_Sheet_1.PDF]

Sw780 DMSO vs. SAHA
Upregulated

Gene Symbol	Description	Log2 Fold Change
SLC17A7	solute carrier family 17 member 7	3.416933054
DHRS2	dehydrogenase/reductase 2	3.414885407
COL5A3	collagen type V alpha 3 chain	3.390427348
CRISPLD2	cysteine rich secretory protein LCCL domain containing 2	3.37563636
C14orf37	chromosome 14 open reading frame 37	3.375371765
HAP1	huntingtin associated protein 1	3.356417573
JUP	junction plakoglobin	3.348715287
DACT3	dishevelled binding antagonist of beta catenin 3	3.304749387
F11R	F11 receptor	3.297755363
TEX15	testis expressed 15	3.296180915
MICA	MHC class I polypeptide-related sequence A	3.291294079
BAIAP3	BAI1 associated protein 3	3.269894422
VWA5B2	von Willebrand factor A domain containing 5B2	3.262456423
SORBS1	sorbin and SH3 domain containing 1	3.261997606
RAB3A	RAB3A, member RAS oncogene family	3.26157698
ABAT	4-aminobutyrate aminotransferase	3.260617415
NID2	nidogen 2	3.256283373
PLEKHH2	pleckstrin homology, MyTH4 and FERM domain containing H2	3.255544506
RAET1G	retinoic acid early transcript 1G	3.248062183
BMF	Bcl2 modifying factor	3.244226813
TSPEAR-AS1	TSPEAR antisense RNA 1	3.235822159
CYP4F22	cytochrome P450 family 4 subfamily F member 22	3.233637055
ULBP2	UL16 binding protein 2	3.224346555
H1F0	H1 histone family member 0	3.204920361
PARM1	prostate androgen-regulated mucin-like protein 1	3.191034304
HSPA1A	heat shock protein family A (Hsp70) member 1A	3.18732935
REEP6	receptor accessory protein 6	3.186892122
CDS1	CDP-diacylglycerol synthase 1	3.161983749
MAN1C1	mannosidase alpha class 1C member 1	3.160066709
TNFRSF19	TNF receptor superfamily member 19	3.157474509

RAI2	retinoic acid induced 2	3.155428557
CACNA1G	calcium voltage-gated channel subunit alpha1 G	3.154293874
C7orf57	chromosome 7 open reading frame 57	3.14656714
SEPT4	septin 4	3.144814623
SERPINE2	serpin family E member 2	3.139285752
DNAJC12	DnaJ heat shock protein family (Hsp40) member C12	3.138424594
PCDH1	protocadherin 1	3.133404246
FAM46C	family with sequence similarity 46 member C	3.131306634
NLRP5	NLR family pyrin domain containing 5	3.129372093
WDR63	WD repeat domain 63	3.119383517
TJP3	tight junction protein 3	3.117587337
TPPP	tubulin polymerization promoting protein	3.11413769
CORO2A	coronin 2A	3.103834772
BICDL1	BICD family like cargo adaptor 1	3.101842134
GPC2	glypican 2	3.082439997
SERPINI1	serpin family I member 1	3.078515679
CORO1A	coronin 1A	3.078028153
RFPL4A	ret finger protein like 4A	3.076126827
PMEL	premelanosome protein	3.070927377
SYT11	synaptotagmin 11	3.065481268
RASD2	RASD family member 2	3.061307792
SLC2A4	solute carrier family 2 member 4	3.05824692
PHOSPHO1	phosphoethanolamine/phosphocholine phosphatase	3.0539519
TNFSF9	tumor necrosis factor superfamily member 9	3.051231705
HRH3	histamine receptor H3	3.049787868
COX6B2	cytochrome c oxidase subunit 6B2	3.049309663
ITGA7	integrin subunit alpha 7	3.046264974
TMEM59L	transmembrane protein 59 like	3.045006533
CTGF	connective tissue growth factor	3.04237426
TUBB4A	tubulin beta 4A class IVa	3.020602638
CRYM	crystallin mu	3.019699581
NCAN	neurocan	3.016743101
CSF1R	colony stimulating factor 1 receptor	3.015716788

TIMP3	TIMP metallopeptidase inhibitor 3	2.997239028
CHST1	carbohydrate sulfotransferase 1	2.993867933
DENND2C	DENN domain containing 2C	2.991514844
KIF5C	kinesin family member 5C	2.979530472
CPM	carboxypeptidase M	2.976427691

Downregulated

Gene Symbol	Description	Log2 Fold Change
LOC101929475	uncharacterized LOC101929475	-2.400746659
PRDM11	PR/SET domain 11	-2.410115467
FOXF2	forkhead box F2	-2.418152014
LINC01444	long intergenic non-protein coding RNA 1444	-2.418517696
SLC16A9	solute carrier family 16 member 9	-2.44749662
TGFB1I1	transforming growth factor beta 1 induced transcript 1	-2.507995379
CLCF1	cardiotrophin-like cytokine factor 1	-2.511715622
LINC01089	long intergenic non-protein coding RNA 1089	-2.552642582
NLRP3	NLR family pyrin domain containing 3	-2.562984397
HMGA2	high mobility group AT-hook 2	-2.564355015
LINC01443	long intergenic non-protein coding RNA 1443	-2.573812364
BDKRB1	bradykinin receptor B1	-2.588697032
MMP14	matrix metallopeptidase 14	-2.598847419
LRFN4	leucine rich repeat and fibronectin type III domain containing 4	-2.610700062
WNT5B	Wnt family member 5B	-2.707736694
CSF1	colony stimulating factor 1	-2.734594397
KCTD15	potassium channel tetramerization domain containing 15	-2.824935124
PLAU	plasminogen activator, urokinase	-2.858079572
CYB561	cytochrome b561	-2.87699786
